# Mutations in gliclazide‐associated genes may predict poor bladder cancer prognosis

**DOI:** 10.1002/2211-5463.12583

**Published:** 2019-02-06

**Authors:** Weiheng Wen, Jinru Gong, Peili Wu, Min Zhao, Ming Wang, Hong Chen, Jia Sun

**Affiliations:** ^1^ Department of Endocrinology Zhujiang Hospital Southern Medical University Guangzhou China; ^2^ State Key Laboratory of Respiratory Disease The First Affiliated Hospital of Guangzhou Medical University China; ^3^ Department of Traditional Chinese Medicine Zhujiang Hospital Southern Medical University Guangzhou China

**Keywords:** bioinformatics, bladder cancer, diabetes, gliclazide

## Abstract

In recent years, an increasing number of patients have had diabetes and cancer simultaneously; thus, it is crucial for physicians to select hypoglycemic drugs with the lowest risk of inducing cancer. Gliclazide is a widely used sulfonylurea hypoglycemic drug, but its cancer risk remains controversial. Here, we explored the primary targets of gliclazide and its associated genes by querying an available database to construct a biological network. By using DrugBank and STRING, we found two primary targets of gliclazide and 50 gliclazide‐associated genes, which were then enrolled for Kyoto Encyclopedia of Genes and Genomes (KEGG) enrichment analysis using WebGestalt. From this analysis, we obtained the top 15 KEGG pathways. Accurate analysis of these KEGG pathways revealed that two pathways, one linked to bladder cancer and the other linked to the phosphoinositide 3‐kinase–AKT signaling pathway, are functionally associated with gliclazide, and from these we identified four overlapping genes. Finally, genomic analysis using cBioPortal showed that genomic alterations of these four overlapping genes predict poor prognosis for patients with bladder cancer. In conclusion, gliclazide should be used with caution as a hypoglycemic drug for diabetic patients with cancer, especially bladder cancer. In addition, this study provides a functional network analysis to flexibly explore drug interaction systems and estimate their safety.

**Abbreviations**
ABCC8: ATP‐binding cassette transporter subfamily C member 8
KEGG: Kyoto Encyclopedia of Genes and Genomes
PI3K: phosphoinositide 3‐kinase
T2DM: type 2 diabetes
VEGFA: vascular endothelial growth factor A

The prevalence of diabetes and cancer is increasing rapidly worldwide. In 2015, more than 415 million people were identified as diabetes patients, and 90% of these were classified as having type 2 diabetes (T2DM) 1. By 2040, this number is projected to be 642 million 1. It is estimated that more than 80% of diabetes patients live in low‐income and middle‐income countries, but the prevalence of diabetes in every country has been increasing since 1980 2. Meanwhile, the global cancer incidence predicted by the World Health Organization will increase to 22 million in 2032, and more than 60% of these cases will occur in Africa, Asia, and Central and South America 3. Diabetes and cancer are closely interrelated in epidemiology and biology. Both of these two disorders share many of the same risk factors, including obesity, aging, physical inactivity, and irregular diet 3. As a result, an increasing number of patients are experiencing both diabetes and cancer.

Gliclazide, a second‐generation oral sulfonylurea agent, has a strong hypoglycemic effect 4. It can be administered in lower doses and used on a once‐daily basis. Gliclazide increases peripheral glucose utilization through activation of hepatic gluconeogenesis and possibly by increasing insulin receptors and sensitivity in specific tissues 5. After binding of gliclazide to a specific sulfonylurea receptor on the β‐cell plasma membrane, it subsequently activates an effector system of insulin secretion. Gliclazide has also been found to have an extrapancreatic effect and thus enhances the sensitivity of peripheral tissues to insulin, which may be the result of augmented biological effects of post‐insulin receptors 5. The new Dutch guideline for T2DM has taken gliclazide as the preferred second‐line drug. Compared with other sulfonylureas, gliclazide has a low risk of hypoglycemic events, which may be explained by the pattern of insulin secretion. The higher reversibility of gliclazide binding to a specific sulfonylurea receptor on β cells resulted in a lesser pancreatic overstimulation, which is the result of fewer hypoglycemic events 6, 7.

Recently, it has been suggested that the use of anti‐diabetic drugs may affect the risk of cancers in T2DM. The use of metformin, for example, has been reported to reduce the risk of cancer 8, while the use of sulfonylurea on cancer risk is still under debate 9, 10, 11. In a population‐based cohort study, increased cancer‐related mortality was found in patients who used sulfonylurea rather than metformin 12. Another observational cohort study containing 568 patients with T2DM showed that glibenclamide users experienced higher mortality for malignancies than gliclazide users 13. Some studies have indicated that different types of sulfonylurea exert different effects on tumors, and gliclazide tends to have beneficial effects on diabetes patients with tumors 10, 13. However, Piccinni and colleagues found that use of gliclazide in bladder cancer led to more adverse events 11. Apart from the inadequate adjustment for confounders, such as complications caused by diabetes and cancers, different effects of gliclazide on specific cancers might contribute to these inconsistent findings, although the relevant research on this topic is limited.

With the rapid development of global genomics research, such as microarray analysis, proteomics assays, and high‐throughput screening research, integration of these available resources has become a flexible yet effective method to determine the relationship between drugs and diseases. In this study, we used DrugBank to study gliclazide and its drug‐target information to further explore gliclazide's underlying pharmacological effects. Based on the direct targets found by DrugBank and associated genes generated by STRING, we further performed pathway enrichment analysis through WebGestalt; the bladder cancer pathway and phosphoinositide 3‐kinase (PI3K)–AKT pathway were screened, and then seven overlapping genes were identified to further analyze their genomic alterations through the cBioPortal database. Taken together, the aim of our research based on these systematic network studies was to explore the safety profile of gliclazide on patients with specific tumors, such as bladder cancer, which reminds us that more in‐depth studies involving the effects of gliclazide on different types of tumors should be conducted.

## Results

### Characterization of bioactivities of gliclazide by DrugBank and visualization of gliclazide linkage network by STRING

**Figure 1:**
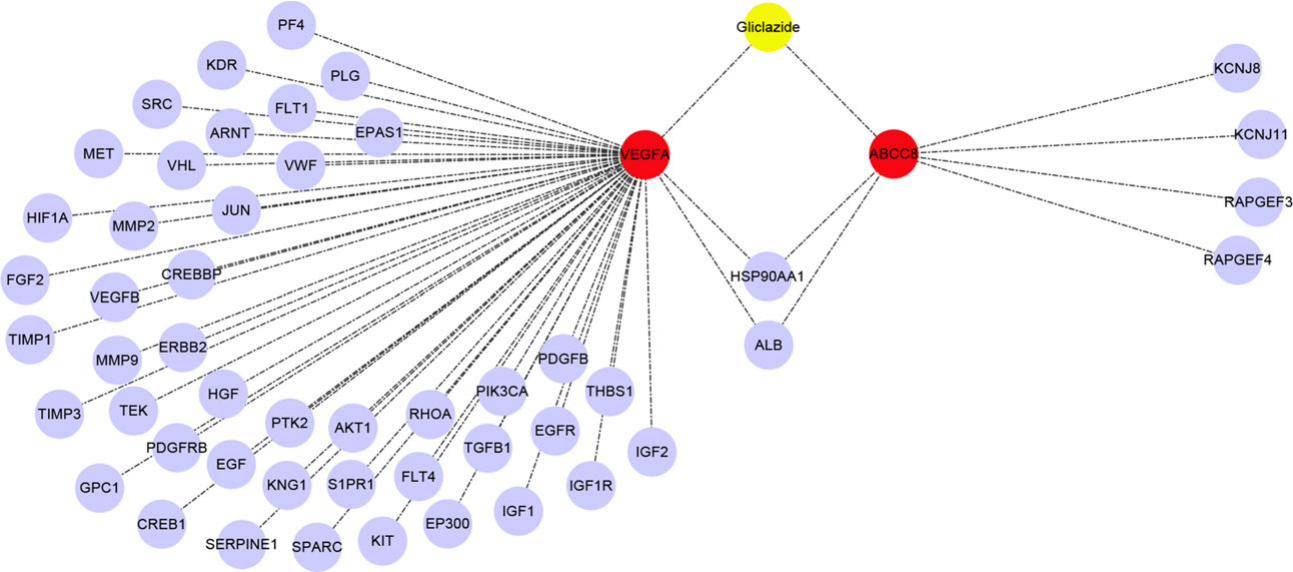
Drug–target interactome of gliclazide. Drug: gliclazide (in yellow); primary direct protein targets: VEGFA and ABCC8 (in red); secondary gliclazide‐associated protein (in purple).

In biosystems, interactions between chemical substances (including drugs or agents), proteins, and gene levels bring a range of biological effects that influence energy homeostasis. To reduce the complexity of analysis, multiple interactions can be visualized as a network. For example, individual nodes can represent biological entities, and the connections between nodes can represent different types of interactions. We first queried DrugBank using gliclazide as an input, which displayed an output of DB01120, categorizing gliclazide as a blood glucose lowering agent, cytochrome P‐450 enzyme inhibitor, sulfonylurea compound, and urea (Table 1). Among these, the main use of gliclazide is to improve glucose metabolism in conjunction with diet and exercise. Table 2 displays two primary direct targets of gliclazide, ATP‐binding cassette transporter subfamily C member 8 (ABCC8) and vascular endothelial growth factor A (VEGFA). To expand our study and analysis, we used STRING to generate a total of 50 gliclazide‐related target proteins (Table S1). This dataset combined with its two primary targets was then collectively integrated to build a biological function network using cytoscape 3.6, thereby displaying a gliclazide network and the visualization of gliclazide‐related protein interactions. The two primary direct targets and their secondary gliclazide‐associated proteins, ABCC8 (five protein targets) and VEGFA (47 protein targets), are shown in Fig. 1. Two genes, *HSP90AA1* and *ALB*, were identified as the common genes linked to both VEGFA and ABCC8. It has been reported that HSP90AA1 is critical to the survival and proliferation of cancer 14. Additionally, the serum level of ALB protein can be used to predict the prognosis of cancer patients 15, 16.

**Table 1:** Characterization of gliclazide using DrugBank

DB_ID	Name	Group	Category	Indication
DB01120	Gliclazide	Approved	Blood glucose lowering agents Cytochrome P‐450 enzyme inhibitors Sulfonylurea compounds Urea	For the treatment of non‐insulin‐dependent diabetes mellitus in conjunction with diet and exercise

**Table 2:** Identification of direct targets of gliclazide using DrugBank

DB_ID	Name	Target	Uniprot ID	Actions	Organism
DB01120	Gliclazide	ABCC8	Q09428	Binder	Human
DB01120	Gliclazide	VEGFA	P15692	Other/unknown	Human

### Analysis of functional features related to gliclazide‐mediated changes in gene set by WebGestalt

To explore the functional attributes of gliclazide‐mediated gene sets, two direct targets found by DrugBank and 50 gliclazide‐related genes generated by STRING were used for KEGG pathway analysis using WebGestalt. The top 15 enrichment pathways associated with gliclazide include epidermal growth factor receptor (EGFR) tyrosine kinase inhibitor resistance (15 genes), Rap1 signaling pathway (23 genes), hypoxia inducible factor‐1 (HIF‐1) signaling pathway (17 genes), PI3K–Akt signaling pathway (23 genes), focal adhesion (22 genes), pathways in cancer (28 genes), proteoglycans in cancer (22 genes), renal cell carcinoma (14 genes), Ras signaling pathway (19 genes), prostate cancer (13 genes), melanoma (11 genes), endocrine resistance (11 genes), bladder cancer (eight genes), cytokine–cytokine receptor interaction (14 genes), and breast cancer (11 genes). All identified biologically effective pathways were statistically significant and thereby warranted further exploration. Broad grouping of the biological function investigation showed that gliclazide‐related genes are closely associated with cancer and its related signal pathway cascades, including (a) regulation of cancer cell proliferation via the tyrosine kinase inhibitor resistance pathway, (b) regulation of tumor growth, invasion, metastasis, and treatment resistance via the HIF‐1 signaling pathway, and (c) regulation of cancer signaling cascades via the PI3K–Akt pathway.

The role of gliclazide in cancer remains controversial, and there is limited research on the effects of gliclazide on site‐specific cancer types. Therefore, more studies should be conducted to evaluate the safety profile of gliclazide on different types of cancers. It has been reported that use of gliclazide in bladder cancer patients caused more adverse events 11, and thus, the bladder cancer pathway was screened for further analysis. Additionally, the Cancer Genome Atlas Research Network found that three major signaling pathways were dysregulated frequently in bladder cancer, namely cell cycle regulation, chromatin remodeling, and PI3K signaling 17. It is generally believed that the P13K pathway plays an important part in the development of bladder cancer 18, 19. Also, KEGG enrichment analysis showed that the PI3K–Akt pathway is tightly related to the gliclazide‐associated network. It seems that gliclazide is more likely to affect bladder cancer development through this pathway. Accordingly, we select the intersection of two gene sets, namely the bladder cancer and PI3K–Akt pathways, which then generates four common genes, *EGF*,* EGFR*,* THBS1*, and *VEGFA* (EntrezGene ID: 1950, 1956, 7057, 7422). Notably, the identified *VEGFA* gene is the direct target of gliclazide. VEGFA is a member of the growth factor family and mediates angiogenesis 20. Several studies have also reported that VEGFA can activate bladder cancer progression 21, 22.

### Mining genomic alterations related to gliclazide‐associated genes in bladder cancer using cBioPortal

**Figure 2:**
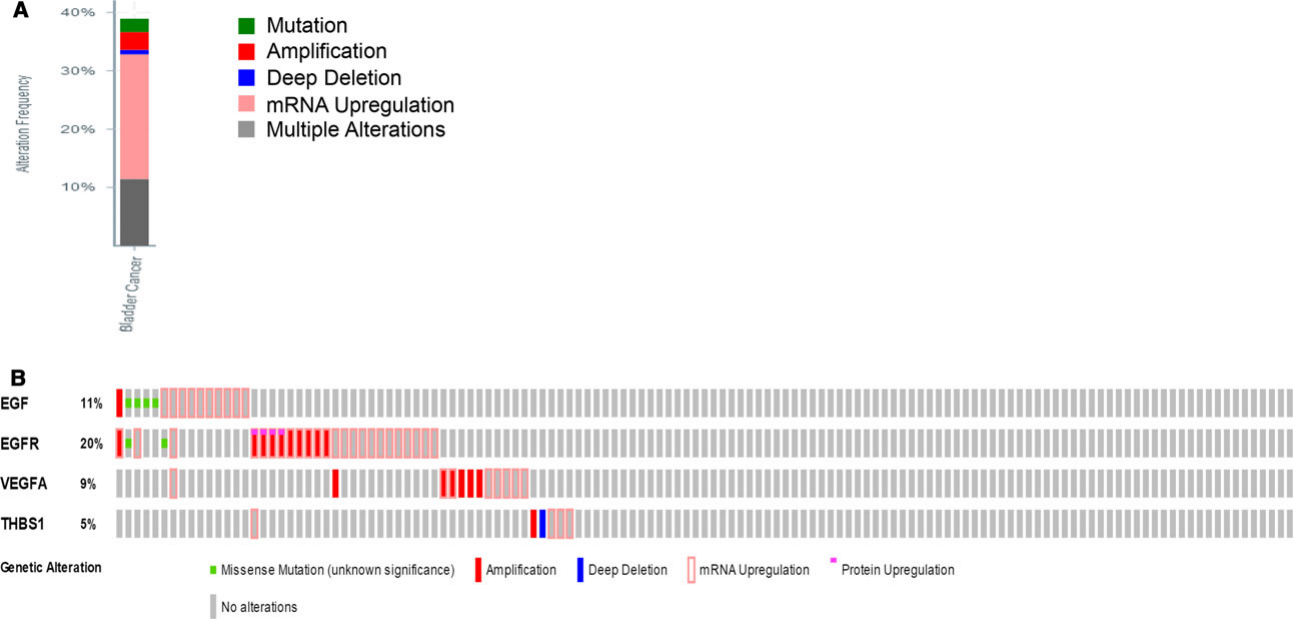
Exploring genetic alterations linked to gliclazide‐associated genes *EGF*,*EGFR*,*VEGFA*, and *THBS1* in bladder cancer using cBioPortal. (A) Overview of changes in *EGF*,*EGFR*,*VEGFA*, and *THBS1* genes in genomic database across a set of bladder cancer samples (based on 17). (B) Oncopoint: a visual display of genomic alteration based on the four identified genes (*EGF*,*EGFR*,*VEGFA*, and *THBS1*). Different genomic alterations are summarized and presented as percentage changes in specific genes. Each row is taken as a gene, and each column is regarded as a sample. Bars of different colors represent different genomic alterations.

To further explore the link between gliclazide‐associated genes and the bladder pathway, cBioPortal was used to uncover genomic alterations of gliclazide‐associated genes in bladder cancer. A summary of seven bladder cancer studies was included in cBioPortal 17, 23, 24, 25, 26, 27. One study was excluded for being accepted provisionally. In order to comprehensively and accurately evaluate the genomic alterations in bladder cancer, a gene set containing four identified genes (*EGF*,* EGFR*,* THBS1*, and *VEGFA*) performed as in the study of The Cancer Genome Atlas Research Network 17, whose genomic profiles included additional mRNA and protein expression levels compared to the other six studies. The results showed that 51 cases (38.93%) have alterations in all four genes (Fig. 2A); the frequency of alteration of four identified genes is shown in Fig. 2B. For *EGFR* (20%), most alterations were mRNA upregulation and amplification, with a few cases of missense mutation and protein upregulation. For *EGF* (11%), the majority of alterations were mRNA upregulation, with a small fraction of amplification and missense mutation. Gene changes associated with *VEGFA* were amplification and mRNA upregulation, and changes associated with *THBS1* were amplification, mRNA upregulation, and deep deletion.

**Figure 3:**
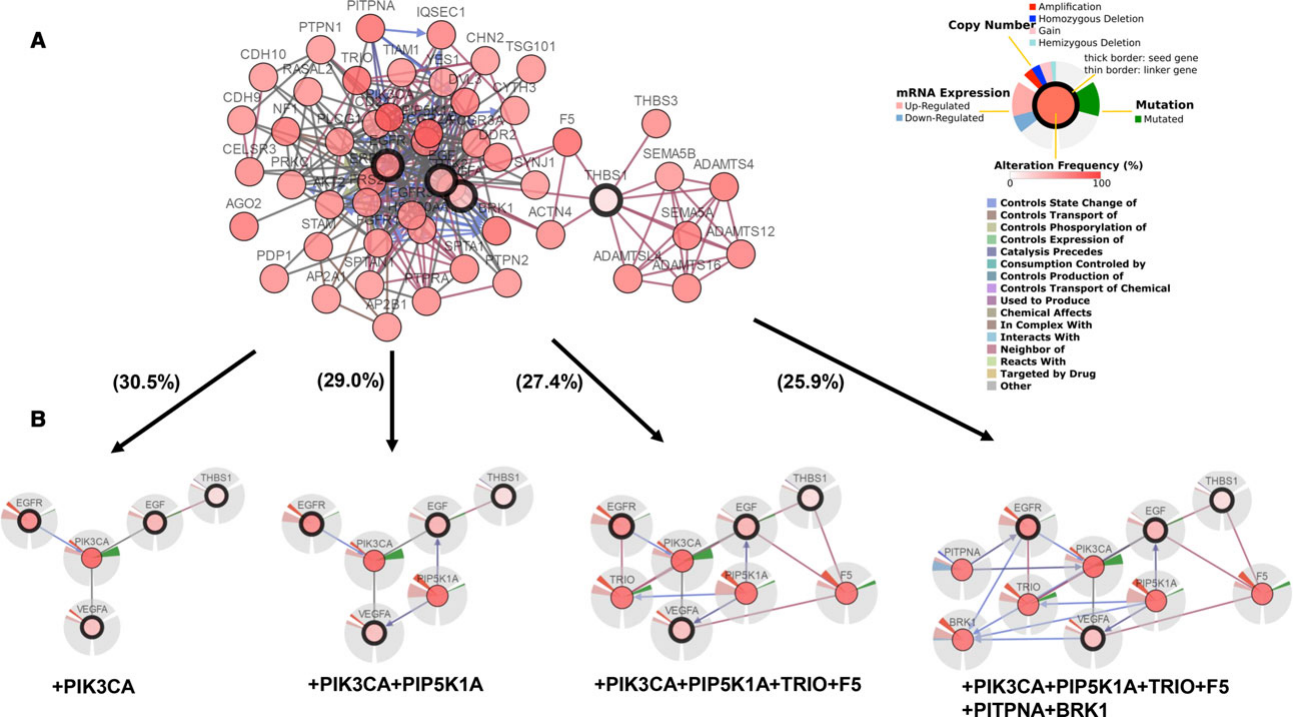
A visual presentation of gene networks linked to *EGF*/*EGFR*/*VEGFA*/*THBS1* in bladder cancer (based on 17). (A) Four selected genes and gliclazide‐associated genes were used as seed genes (identified with a thick black border) to explore all other genes that were altered in bladder cancer samples using cBioPortal. (B) Neighboring genes linked to the four identified genes were filtered by alteration (%). Darker red represents increased frequency of alterations in bladder cancer. The filter applied within the selected bladder cancer study contained the highest genomic alteration frequency in addition to the selected genes.

cBioPortal also can be used to analyze the interactive analysis and construct networks which are altered in bladder cancer. We first built a network including all neighbors of four identified genes, *EGF*,* EGFR*,* THBS1*, and *VEGFA* (Fig. 3). Next, we took the genomic alteration frequency within the screened bladder cancer study as a filter such that only the genes with high alteration frequency were displayed, thereby reducing the complexity of the analysis. First, the four selected genes were identified to be associated with PIK3CA using a filter of 30.5% alteration. Comparatively, five genes, including *PIP5K1A*, were displayed using a filter of 29.0% alteration. A cluster of eight genes, including *PIK3CA*,* PIP5K1A*,* TRIO*, and *F5*, were shown after the filter was decreased to 27.4%, while 10 genes, including *PIK3CA*,* PIP5K1A*,* TRIO*,* F5*,* PITPNA*, and *BRK1*, were demonstrated with a filter of 25.9%. The integrated and pruned networks showed the underlying interactions and the variability of differences between gliclazide‐associated genes and the bladder cancer samples in the study of 17 (Fig. 3).

**Figure 4:**
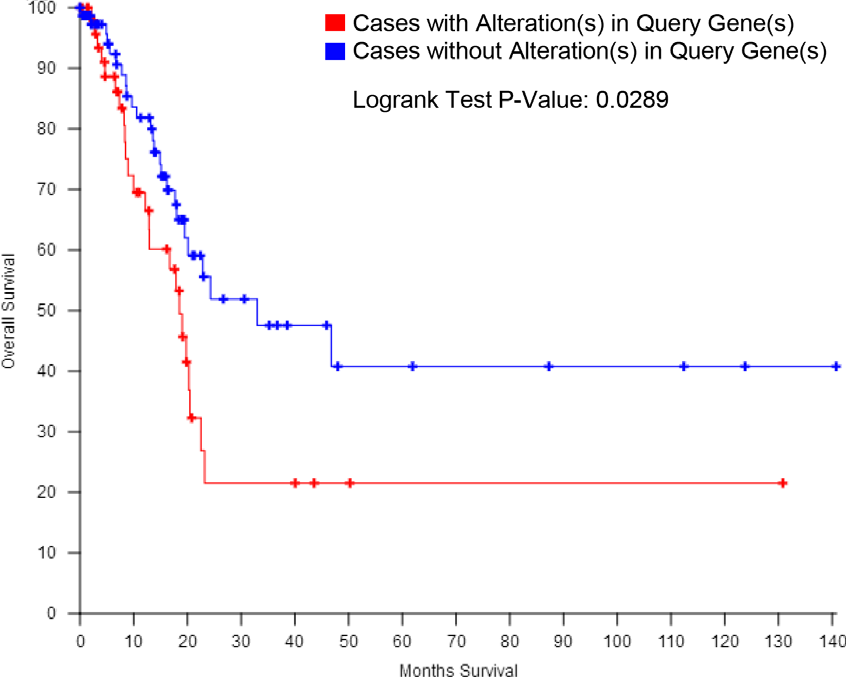
Survival analysis according to the genomic alterations of *EGF*/*EGFR*/*VEGFA*/*THBS1* in bladder cancer. The genomic alterations of the four identified genes were associated with a significant reduction in overall survival rate.

To further explore the association between genomic alterations of gliclazide‐associated genes and the survival of patients with bladder cancer, the four identified genes (*EGF*,* EGFR*,* THBS1*, and *VEGFA*) were used to perform a survival analysis as in the study of The Cancer Genome Atlas Research Network 17. The result showed that patients with alterations in four identified genes had a lower overall survival rate (*P* = 0.0289) (Fig. 4).

## Discussion

Type 2 diabetes is a growing global health problem, which results in serious acute and chronic complications that affect the quality of people's lives. Current epidemiological evidence indicates that diabetes increases the risk of cancers, and the possible biological mechanisms may be associated with hyperinsulinemia, hyperglycemia, and chronic inflammation 28. As such, an increasing number of patients have diabetes and cancer at the same time, making it essential for patients to use hypoglycemic drugs that have no effect on cancer treatment. To our knowledge, the impact of sulfonylurea drugs on cancer has not yet been determined, but some studies have suggested that this result may be caused by different effects of different sulfonylurea drugs on cancer, such as the benefit of the use of gliclazide for cancer 10, 29. However, gliclazide also has been reported to cause more side effects in bladder cancer patients 11. Apart from not excluding complex confounding factors, limited research on gliclazide has been performed in specific cancer types contributing to these inconsistent results. Therefore, a new analytical technique and platforms are required to assess the effect of gliclazide on specific types of cancer.

In this study, we used a set of web‐based tools to analyze the functional network of gliclazide, which also helped to eliminate the interference of confounding factors in clinical research. We explored the molecular action of gliclazide by querying cancer genomics data in three open platforms, DrugBank, STRING, and cBioPortal. This flow of work contains two simple procedures: (a) exploring the direct targets of gliclazide and functional network with gliclazide‐associated protein using DrugBank and STRING, and (b) identifying whether genomic alterations of gliclazide‐associated genes exist in the sample in cancer genomic projects using cBioPortal. This study helped us to interactively explore the effective targets of gliclazide, further explore the biological effect pathways based on the target and gliclazide‐associated genes, and finally connect to the clinical results formulated by mining multiple integrated web‐based databases. Also, this approach, with significant biological rationality, can not only effectively integrate and use existing resources, but also reduce unnecessary experiments and give better direction for scientific research.

In this study, we constructed a functional network to illustrate the connectivity between gliclazide and cancer using open integrated databases. Analysis of gliclazide through these accessible platforms identified two primary target proteins (ABCC8 and VEGFA), another 50 gliclazide‐related proteins and the enriched KEGG pathway closely associated with gliclazide (Fig. 1). In accordance with the known functional features with gliclazide‐related genes and KEGG pathway analysis, we found that 28 genes were associated with cancers in humans (Table 3). This biological effect may be initiated by the interaction between gliclazide and its target proteins. As previously described, the effect of gliclazide on cancer remains controversial. Piccinni and colleagues reported that use of gliclazide caused a series of adverse events in patients with bladder cancer 11. Our KEGG pathway analysis also showed that gliclazide‐related genes are associated with bladder cancer (Table 3). As such, we determined to explore the connectivity between gliclazide and bladder cancer. Additionally, the PI3K–Akt signal pathway, which is critical in the development of bladder cancer 18, 19, is tightly linked to the gliclazide‐associated network revealed by KEGG analysis (Table 3). This suggested that the use of gliclazide affects bladder cancer development through the PI3K–AKT pathway. Therefore, four overlapping genes (*EGF*,* EGFR*,* VEGFA*, and *THBS1*) were extracted from the bladder cancer and PI3K–AKT pathways. Notably, VEGFA is the primary target of gliclazide (Fig. 1). Next, we further explored the genomic alterations of gliclazide‐associated genes in bladder cancer using cBioPortal. In bladder cancer patients, the majority of *EGF* and *EGFR* alterations are mRNA upregulation and amplification (Fig. 2), which led to an upregulation of their expression associated with the progression in bladder cancer 30, 31, 32. In terms of *VEGFA*, the most obvious change was amplification (Fig. 2), which promotes the expression of *VEGFA* in accordance with the development and poor prognosis in bladder cancer 33, 34. Furthermore, our results also revealed that patients with alterations in the four identified genes showed a lower overall survival rate (Fig. 4). This may offer a therapeutic insight into the use of gliclazide in T2DM with bladder cancer.

**Table 3:** List of enriched gliclazide‐related gene sets using WebGestalt. *C*, the number of genes referenced in a specific category; *O*, the number of genes that overlap both in gene set and category; *E*, expected number of genes within the category; *R*, the ratio of enrichment analysis; *P*, calculated by the hypergeometric test

Pathway name	No. of genes	Gene (corresponding gene set)	Statistics
EGFR tyrosine kinase inhibitor resistance	15	*EGF*,* EGFR*,* ERBB2*,* AKT1*,* FGF2*,* HGF*,* IGF1*,* IGF1R*,* KDR*,* MET*,* PDGFB*,* PDGFRB*,* PIK3CA*,* SRC*,* VEGFA*,* PDGFRB*,* PIK3CA*,* SRC*,* VEGFA*	*C* = 81; *O* = 15; *E* = 0.58; *R* = 26.05; *P* = 0
Rap1 signaling pathway	23	*RAPGEF3*,* RAPGEF4*,* EGF*,* EGFR*,* AKT1*,* FGF2*,* FLT1*,* FLT4*,* HGF IGF1*,* I GF1R*,* KDR*,* KIT*,* RHOA*,* MET*,* PDGFB*,* PDGFRB*,* PIK3CA*,* SRC*,* TEK*,* THBS1*,* VEGFA*,* VEGFB*	*C* = 212; *O* = 23; *E* = 1.51; *R* = 15.26; *P* = 0
HIF‐1 signaling pathway	17	*CREBBP*,* EGF*,* EGFR*,* EP300*,* ERBB2*,* AKT1*,* FLT1*,* HIF1A*,* IGF1*,* IGF1R*,* ARNT*,* SERPINE1*,* PIK3CA*,* TEK*,* TIMP1*,* VEGFA*,* VHL*	*C* = 103; *O* = 17; *E* = 0.73; *R* = 23.22; *P* = 0
PI3K‐Akt signaling pathway	23	*CREB1*,* EGF*,* EGFR*,* AKT1*,* FGF2*,* FLT1*,* FLT4*,* HGF*,* HSP90AA1*,* IGF1*,* IGF1R*,* KDR*,* KIT*,* MET*,* PDGFB*,* PDGFRB*,* PIK3CA*,* PTK2*,* TEK*,* THBS1*,* VEGFA*,* VEGFB*,* VWF*	*C* = 341; *O* = 23; *E* = 2.42; *R* = 9.49; *P* = 0
Focal adhesion	22	*EGF*,* EGFR*,* ERBB2*,* AKT1*,* FLT1*,* FLT4*,* HGF*,* IGF1*,* IGF1R*,* JUN*,* KDR*,* RHOA*,* MET*,* PDGFB*,* PDGFRB*,* PIK3CA*,* PTK2*,* SRC*,* THBS1*,* VEGFA*,* VEGFB*,* VWF*	*C* = 203; *O* = 22; *E* = 1.44; *R* = 15.25; *P* = 0
Pathways in cancer	28	*CREBBP*,* EGF*,* EGFR*,* EP300*,* EPAS1*,* ERBB2*,* AKT1*,* FGF2*,* HGF*,* HIF1A*,* HSP90AA1*,* IGF1*,* IGF1R*,* JUN*,* KIT*,* RHOA*,* ARNT*,* MET*,* MMP2*,* MMP9*,* PDGFB*,* PDGFRB*,* PIK3CA*,* PTK2*,* TGFB1*,* VEGFA*,* VEGFB*,* VHL*	*C* = 397; *O* = 28; *E* = 2.82; *R* = 9.92; *P* = 0
Proteoglycans in cancer	22	*EGFR*,* ERBB2*,* AKT1*,* FGF2*,* GPC1*,* HGF*,* HIF1A*,* IGF1*,* IGF1R*,* IGF2*,* KDR*,* RHOA*,* MET*,* MMP2*,* MMP9*,* PIK3CA*,* PTK2*,* SRC*,* TGFB1*,* THBS1*,* TIMP3*,* VEGFA*	*C* = 205; *O* = 22; *E* = 1.46; *R* = 15.10; *P* = 0
Renal cell carcinoma	14	*CREBBP*,* EP300*,* EPAS1*,* AKT1*,* HGF*,* HIF1A*,* JUN*,* ARNT*,* MET*,* PDGFB*,* PIK3CA*,* TGFB1*,* VEGFA*,* VHL*	*C* = 67; *O* = 14; *E* = 0.48; *R* = 29.40; *P* = 0
Ras signaling pathway	19	*EGF*,* EGFR*,* AKT1*,* FGF2*,* FLT1*,* FLT4*,* HGF*,* IGF1*,* IGF1R*,* KDR*,* KIT*,* RHOA*,* MET*,* PDGFB*,* PDGFRB*,* PIK3CA*,* TEK*,* VEGFA*,* VEGFB*	*C* = 229; *O* = 19; *E* = 1.63; *R* = 11.67; *P* = 3.33E‐16
Prostate cancer	13	*CREB1*,* CREBBP*,* EGF*,* EGFR*,* EP300*,* ERBB2*,* AKT1*,* HSP90AA1*,* IGF1*,* IGF1R*,* PDGFB*,* PDGFRB*,* PIK3CA*	*C* = 89; *O* = 13; *E* = 0.63; *R* = 20.55; *P* = 2.16E‐14
Melanoma	11	*EGF*,* EGFR*,* AKT1*,* FGF2*,* HGF*,* IGF1*,* IGF1R*,* MET*,* PDGFB*,* PDGFRB*,* PIK3CA*	*C* = 71; *O* = 11; *E* = 0.50; *R* = 21.80; *P* = 1.39E‐12
Endocrine resistance	11	*EGFR*,* ERBB2*,* AKT1*,* IGF1*,* IGF1R*,* JUN*,* MMP2*,* MMP9*,* PIK3CA*,* PTK2*,* SRC*	*C* = 98; *O* = 11; *E* = 0.70; *R* = 15.79; *P* = 5.28E‐11
Bladder cancer	8	*EGF*,* EGFR*,* ERBB2*,* MMP2*,* MMP9*,* SRC*,* THBS1*,* VEGFA*	*C* = 41; *O* = 8; *E* = 0.29; *R* = 27.45; *P* = 2.94E‐10
Cytokine‐cytokine receptor interaction	14	*EGF*,* EGFR*,* FLT1*,* FLT4*,* HGF*,* KDR*,* KIT*,* MET*,* PDGFB*,* PDGFRB*,* PF4*,* TGFB1*,* VEGFA*,* VEGFB*	*C* = 265; *O* = 14; *E* = 1.88; *R* = 7.43; *P* = 2.37E‐09
Breast cancer	11	*EGF*,* EGFR*,* ERBB2*,* AKT1*,* FGF2*,* FLT4*,* IGF1*,* IGF1R*,* JUN*,* KIT*,* PIK3CA*	*C* = 146; *O* = 11; *E* = 1.04; *R* = 10.60; *P* = 4.02E‐09

The use of gliclazide for site‐specific types of cancer remains unclear. Some studies suggest that gliclazide can bring benefit to cancer patients because of its antioxidant effect 10. However, other studies suggest that using gliclazide is not conducive to the treatment of cancers. Sliwinska and colleagues showed that the anti‐oxidation effect of gliclazide does not merely protect normal cells against apoptosis, but also makes cancer cells resistant to apoptosis 35. Meanwhile, gliclazide also has been reported to activate DNA repair in cancer cells rather than normal human cells 36. Therefore, more reasonable and standardized clinical research and basic scientific research are needed to clarify the relationship between gliclazide and cancer. The biological network and pathway analysis we have constructed allows us to achieve this. The enriched KEGG pathway analysis also shows that gliclazide has the potential to affect renal and prostate cancer – an association which has not received enough attention from researchers (Table 3). Thus, this approach can broaden our understanding of malignancy and help in early diagnosis and prognosis of disease. Also, mining and analyzing existing data based on open databases can not only provide relevant information such as drug safety, but also find a basis for drug repurposing and thereby reduce the cost of drug development.

Certain limitations within this analysis should be acknowledged. First, the validation of protein expression may better reveal the biological function of gliclazide, and this was actually not available in our study. The nature of the interactions needs further exploration to investigate the underlying mechanism by which gliclazide causes more side effects in bladder cancer patients. Second, other specific types of cancer pathways presented by enrichment analysis have not been explored. Whether the biological function network between gliclazide and bladder cancer displayed in this research can be extended to other cancers, such as renal and prostate cancer, remains for further exploration. However, the results of this research may provide the basis for further experimental work and help investigators translate basic study into clinical applications.

In conclusion, we integrated and used the open information databases to build a biological network to explore the relationship between gliclazide and cancer. As an increasing number of studies are conducted, more gliclazide‐targeted proteins will be discovered in the future, which will help us better understand the mechanisms involved. We believe that this method can be conducted to guide basic research and clinical applications. For example, our study showed that genomic alterations of gliclazide‐associated genes predict a poor prognosis for patients with bladder cancer. Therefore, for diabetes patients with cancer, especially bladder cancer, doctors should seriously consider whether they should choose hypoglycemic drugs with lower risks rather than gliclazide. Overall, this study provides a simple yet flexible approach to test the reasonable presumption of genetic alterations in bladder cancer and relevant information regarding drug safety by applying available drug information.

## Materials and methods

### Drug‐target search

The DrugBank database is an available bioinformatics and cheminformatics tool able to query detailed drug data and provide comprehensive target information 37, 38. This database contains 11 033 drug entries, consisting of 2521 approved small molecules, 950 biotech drugs, 111 nutraceuticals, and over 5112 experimental drugs. The data are analyzed, grouped, and then used to establish a drug–target interactome. In this research, DrugBank was used to explore the indications and category for gliclazide. Additionally, the interaction between gliclazide and its targets was then generated for further construction of a gliclazide‐associated biological network. These data were used to establish a visualization work following further analysis as well as to develop a proposal for future experiments.

### Network generation and pathway enrichment analysis

STRING version 10.5 is a database of known and predicted protein–protein interactions, which includes direct (physical) and indirect (functional) associations 39. The interaction information was generated from computational prediction and knowledge transfer between organisms as well as other (primary) databases. In this study, target proteins of gliclazide were obtained using the STRING online database, and the results were further visualized using cytoscape (version 3.6.0) 40. WebGestalt is an integrated functional enrichment analysis database that offers flexible and accurate analysis of functional enrichment pathways 41. Gliclazide‐associated genes were queried into the WebGestalt database, which resulted in the top 15 Kyoto Encyclopedia of Genes and Genomes (KEGG) enrichment pathways with *P*‐values < 0.01.

### Cancer genomics data linked to gliclazide and bladder cancer survival analysis

The cBioPortal is an open web‐based database for exploring multidimensional cancer genomics information obtained from cancer samples 42, 43. Complicated genomic information is easily accessible enabling researchers to analyze their genetic alterations across cancer samples from various studies. The relevant information obtained is closely linked to the clinical outcomes, which is conducive to further exploring new connections. In this study, the cBioPortal database was used to analyze the connectivity of gliclazide‐associated genes across bladder cancer studies, and these genes were taken as altered or unaltered in bladder cancer samples. Finally, the altered genes were used to perform a survival analysis using the cBioPortal database.

## Conflict of interest

The authors declare no conflict of interest.

## Author contributions

HC and JS conceived and designed the study; WW, JG and PW analyzed data and wrote the manuscript; MZ participated in data analysis; MW help modify the manuscript and provided suggestions. All authors read and approved the final manuscript.

## Supporting information


**Table S1.** Fifty gliclazide‐related genes.
